# Description of Position Ads for Pharmacy Technicians

**DOI:** 10.3390/pharmacy8020088

**Published:** 2020-05-22

**Authors:** Juanita A. Draime, Emily C. Wicker, Zachary J. Krauss, Joel L. Sweeney, Douglas C. Anderson

**Affiliations:** 1 Department of Pharmacy Practice, Cedarville University School of Pharmacy, Cedarville, OH 45413, USA; andersond@cedarville.edu; 2 Cedarville University School of Pharmacy, Cedarville, OH 45413, USA; ewicker217@cedarville.edu (E.C.W.); zacharykrauss@cedarville.edu (Z.J.K.); jsweeney@cedarville.edu (J.L.S.)

**Keywords:** pharmacy technician, technician duties, technician job requirements, technician role

## Abstract

Pharmacy technician roles are evolving alongside the changing role of a pharmacist. There is currently no uniform definition of a pharmacy technician’s role in the pharmacy workforce. The objective of this study was to look at the United States-based pharmacy technician advertisement database from Pharmacy Week to find patterns and commonalities in the duties and qualifications of pharmacy technicians. A retrospective analysis was performed on fourteen days of pharmacy technician job listings from Pharmacy Week from the year 2018. Information obtained from the listings included job title, location, setting, type of job, job duties, and job requirements. Job duties and requirements were coded by themes. Fourteen days of data resulted in 21,007 individual position listings. A majority of the job listings were for full-time positions (96.4%) and most were in the retail setting (96.78%). The most common requirements were registration with State Board, high school diploma, ability to perform tasks, communication, and physical. The most common job duties were general office etiquette, performing tasks under the direct supervision of the pharmacist, and professionalism. This study provides a description of the evolving role of pharmacy technicians through the broad variety in expectations for requirements of pharmacy technician applicants and the duties they perform when hired.

## 1. Introduction

Over the last decade, pharmacy has seen a large shift in the role of both the pharmacist and pharmacy technician. For instance, pharmacists have increasingly focused on providing clinical care, and gradually fallen away from the traditional dispensing role [1]. Pharmacy technicians are personnel “working in a pharmacy who, under the supervision of the licensed pharmacist, assists in pharmacy activities that do not require the professional judgment of a pharmacist [1].” However, as pharmacists have decreased their focus on dispensing, pharmacy technicians have been given the opportunity to fill the traditional dispensing role in the pharmacist’s place. These new changes have progressively become the normal pharmacy model, and many studies have shown the added value of this model in both improved quality of patient care and pharmacy efficiency [2,3,4,5,6,7,8]. This shift is a huge building block for the future, yet at the same time, it has resulted in the formation of unique and evolving roles for pharmacy technicians. These new roles are still in the development process, and research is beginning to roll out on the safety and efficacy of advanced pharmacy technician positions [9,10,11,12]. These changes, in due course, may be considerable innovations, but at the same time, they may pose poignant risks to patient safety. This study helps to define pharmacy technicians’ new roles through the use of pharmacy technician job advertisements.

Understanding the way pharmacy technicians are recruited through job advertisements is helpful in defining the evolving role of pharmacy technicians. The recruitment process through job listings on social forums is an important and commonly-used tool by employers to convey job requirements and responsibilities. Additionally, job advertisements affect the number, type, and talent of possible hires. If effectively used, employers can use job advertisements to increase the quality and quantity of applicants by giving applicants a clear snapshot of future roles and responsibilities as well as the requirements for the job. While advertisements do not represent the formal contractual responsibilities and requirements of a technician, they do represent what the employer needs in a technician. Ultimately, looking at pharmacy technician advertisements can serve as an indicator of both the duties and qualifications desired for pharmacy technicians.

The roles and responsibilities of pharmacy technicians have been evolving from basic pharmacy organization and prescription assembly skills to complex dispensary, verification, immunization, education, and medication synchronization skills in the past decade [13]. Recently, the American Society of Health-System Pharmacists (ASHP) has defined the role of pharmacy technicians in three categories: entry-level, advanced, and specialized [13]. The listed skills needed for entry-level roles include pharmacology for technicians, pharmacy law and regulation, compounding including low- or medium-risk sterile compounding and non-sterile compounding, basic safe medication practices, pharmacy quality assurance, medication order entry and distribution, pharmacy inventory management, pharmacy billing and reimbursement, and medication-use system technology [13]. Interestingly, many of what the ASHP calls “entry-level skills” are what used to traditionally define the role of pharmacy technician [1]. However, as the pharmacy model has evolved, pharmacy technicians are now receiving new roles that are attained through additional education, training, and competency testing. These new advanced roles are supervised by pharmacists and/or approved by each state’s Board of Pharmacy [1]. According to the ASHP, pharmacy technicians need to have certain skills to be included in this advanced category [13]. These skills include advanced medication systems including “tech-check-tech” programs, purchasing or fiscal management, management or supervision of other pharmacy technicians, medication history assistance, medication therapy management assistance, quality improvement, immunization assistance, hazardous drug handling, patient assistance programs, pharmacy technician education and training, community outreach, drug utilization evaluation and/or adverse-drug-event monitoring, industry, and informatics [13]. Furthermore, the ASHP says that some technicians receive even more specialized roles which are dependent upon each technician’s individual situation [13]. They define these specialized roles as roles that require extra certification as specified by the Pharmacy Technician Certification Board (PTCB) [13]. These advanced and specialized roles contain many unique and innovative changes for pharmacy technicians which could ultimately improve patient care [13].

Despite the fact that these new technician roles may help alleviate a pharmacist’s workload and allow for a more streamlined pharmacy, they may, at the same time, present safety and efficacy issues in patient care. For instance, many of these new roles are still in the research and development process and, therefore, require strict certification, regulation, and supervision by each state’s Board of Pharmacy [9,10,11,12,14]. These innovative roles require exemptions as can be seen in the research studies done by Frost, Adams. McKeiran, Henriksen, and Bailey [9,10,11,12,14]. However, employers may knowingly or unknowingly try to utilize the added efficiency of these new roles in their pharmacies without receiving proper exemption status. Unfortunately, without the provided exemption, many of the strict protocols and regulations put into place by Boards of Pharmacy as safeguards may be ignored or improperly implemented. Without proper regulation, there is a higher chance of errors and mistakes. As a result, employers may run into legal issues, and, more importantly, patient safety and efficacy of care will be put at risk. A previous study on pharmacy technician training programs found that of 216 training programs, 29.6% were accredited and 46% had pharmacists as faculty of the program. It was concluded that there is little to no oversight of and consistency in pharmacy technician training [15].

While, this topic should be of great interest to employers, there is a lack of literature studying the hiring requirements and expectations of pharmacy technicians. A review of state regulations concerning entry-level pharmacy technicians in 2017 found that 86% of states required board registration or licensure. While only 16 states required any training programs for entry-level pharmacy technicians. This study reveals the legal requirements for the employment of pharmacy technicians, but did not study or discuss what employers actually look for in pharmacy technician candidates [16]. Therefore, the overall objective of this study was to look at the pharmacy technician advertisement database from the Pharmacy Week to find patterns and commonalities in the duties and qualifications of pharmacy technicians.

## 2. Materials and Methods

This study used a retrospective analysis study design to describe the current pharmacy technician job descriptions. Pharmacy technician job advertisements were obtained from Pharmacy Week for a 14 day period (26 November 2018–9 December 2018). PharmacyWeek.com was an online database of job advertisements for pharmacists, pharmacy technicians, and pharmacy interns started in 1990. Unfortunately, it is no longer maintained and can no longer be accessed. For all pharmacy technician job advertisements, the following items were obtained: (1) job title, (2) location of job (city and state), (3) what field of pharmacy job is in (hospital system, retail, [chain versus independent], long-term care, managed care, etc.), (4) position type (full or part time), (5) job duties, and (6) listed requirements for the position. Requirements within job advertisements were noted as being either required, preferred, not required, or not specified. Duties within job advertisements were noted as being either listed or not listed. Following data collection, related requirements and duties were combined into themes. These themes are detailed in Table 1 and Table 2. The decision to code requirements and duties by themes verses individually was made by all researchers after listing were made of all the individual requirements and duties found in the position listing. The final themes agreed upon are depicted in the Table 1 and Table 2.

Data were collected and entered into an Excel document. Data were de-identified prior to Excel entry. No information was recorded in a manner that could reveal the identity. Descriptive statistics were performed for all data in IBM© SPSS v 26.0 (Armonk, NY, USA). This included frequencies and percentages.

## 3. Results

**Figure 1:**
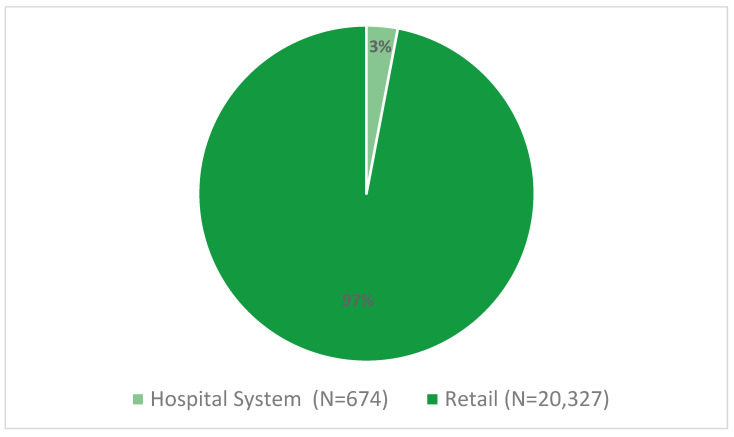
Pharmacy technician job listings by setting.

Fourteen days of data resulted in 21,007 individual position listings, with 96.78% of those being in a retail setting. These technician position listings included positions from all 50 states, Puerto Rico, and the District of Columbia. A little over one-third (37.5%) of the positions were from California, Florida, Illinois, New York, or Texas (N = 1983, 9%; N = 1889, 9%; N = 1242, 6%; N = 1188, 5.7%; and N = 1568, 7.5%, respectively). A majority of the job listings were for full-time positions (96.4%). Settings for these positions included hospital systems, retail pharmacies, and managed care companies (Figure 1).

Pharmacy technician job listing in the managed care setting are not reflected in Figure 1 due to sample size of 6.

The requirements included in the listings are displayed in Table 3. The most common requirements were registration with State Board, high school diploma, ability to perform tasks, communication, and physical (N = 18,261 86.9%, N = 17,325 82.5%, N = 16,861 80.3%, N = 16,436 78.2%, and N = 15,908 75.7%, respectively).

Additional information collected but not reported in Table 3 include that 9.1% (N = 1904) required some form of technician program coursework and almost 1% of listings required or preferred at least an associate’s degree level of education (required N = 131, 0.6%; preferred N = 56, 0.3%).

The job duties included in the listings are displayed in Table 4. The most common job duties were general office etiquette, performing tasks under the direct supervision of the pharmacist, and professionalism (19,961 95%, 18,043 85.9%, and 10,560 50.3%, respectively.

Additional information collected related to duties worth noting include 10 listings that required pharmacy technicians to have experience in patient assistance programs (0.0%, N = 10), HIV knowledge (0.0%, N = 3), maintaining narcotic coordination and investigational drug therapy (0.8%, N = 167), calculations (0.4%, N = 76), and managing difficult or emotional patient situations (0.0%, N = 2).

**Figure 2:**
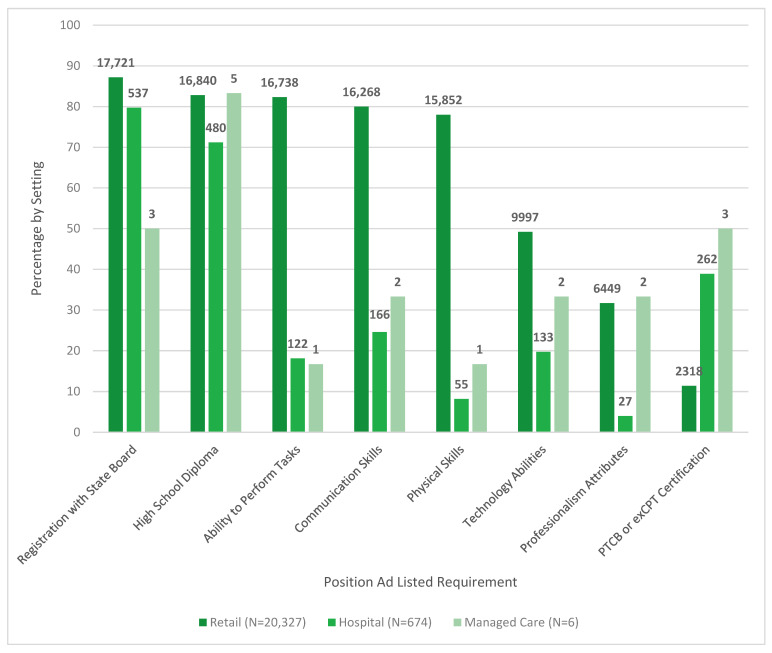
Pharmacy technician position ad requirements by setting.

**Figure 3:**
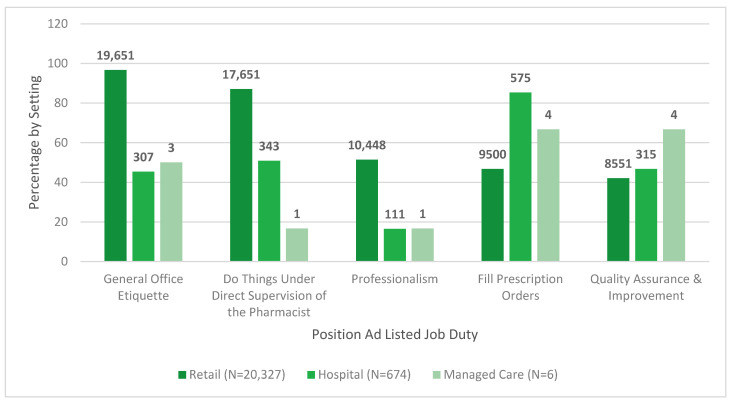
Pharmacy technician position ad job duties by setting.

In Figure 2 and Figure 3, the pharmacy technician position ad requirements and duties are indicated by job setting.

Of note in the pharmacy requirements as separated by setting, 11.7% (N = 79) of included hospital advertisements included a legal piece of some sort, whereas less than 1 % (0.3%, N = 55) of retail and 0 in managed care did.

Some individual technician ads listed unique and unheard of duties and requirements for applicants. One example of this would be a job duty listed as “be HIV knowledgeable” without further context; the same ad listed that technicians were expected to assist patients in “solving issues and problems related to AIDS.” Another example was a pharmacy technician position that expected the applicant to be able to operate a forklift and hand tools. One technician position examined included requirements that technicians be willing to travel for meetings, conferences, and “field support” in order to “support and grow key customer relationships.”

## 4. Discussion

A review of job postings for pharmacy technicians provided a rich description of the different roles and skills currently needed across pharmacy settings. Technicians remain in many traditional settings, such as retail/community pharmacy. This is consistent with other descriptions that place technicians in supportive roles at community pharmacies and health-system pharmacies [1]. In these settings, technicians often assist with technical pharmacy functions and interact with patients. However, this can expand to other roles, such as providing support for medication therapy management (MTM) services and other clinical services [1]. Interestingly, there are emerging areas that are hiring technicians, such as managed care. As pharmacists in managed care continue to expand their opportunities to provide clinical services and chronic care management, technicians may have increasing roles to support data collection and documentation [15].

The data show that 12.3% (N = 2583) of pharmacy technicians are required to have standard certifications in pharmacy technician work such as PTCB and exCPT. Many states have a requirement that pharmacy technicians get certified either through these routes or through a standardized exam with similar content within a year of hiring, but there is little standardization across the board for these exams [17,18]. On multiple occasions, calls for standard national training and certification processes have been made, and it is clear that multiple organizations find it crucial to have this kind of a standard for certified pharmacy technicians [19,20]. While 81.5% (N = 17,114) did prefer a certified technician, it is surprising that more employers do not share the same national desire.

To fill these roles in a variety of settings, pharmacy technicians appear to need a variety of skills—many of which lie in the affective domain, i.e., professionalism and communication. Given the customer/patient service role many technicians provide, it is essential that they exhibit professionalism and can communicate appropriately in both written and verbal formats [2]. Further, analysis of the ads underscored the importance of technicians in quality assurance. Given their role in the dispensing process, inventory management, and other aspects of the pharmacy, technicians play a vital role in fostering an environment that promotes safe and effective medication use. For example, Odukoya, Schleiden, and Chui (2015), found that pharmacy technicians play a vital role in preventing e-prescribing errors by catching errors before the prescription is sent to the pharmacist to verify [16].

Expanding the roles of certified pharmacy technicians continues to be a discussion in the literature and the profession of pharmacy. States continue to explore tech-check-tech programs to free up pharmacist time to focus on clinical activities [9]. Some recent research also has explored the use of pharmacy technicians to extend medication management to the home setting, performing medication reconciliation and reiterating key counseling points from the pharmacist using motivational interviewing and the teach-back method [14]. Others have explored creating a clinical pharmacy technician to expand patient medication education [17]. While the job postings do not necessarily reflect these expansions, it would be important to continue to monitor ads to determine if the roles of pharmacists, and particularly, certified pharmacy technicians, are altering and expanding to allow pharmacists to focus on clinical activities.

As noted in the results, a small portion of the data included advertisements that required the technicians to be knowledgeable in HIV-related patient care. While this was not a substantial portion of the data, it is interesting to note that it is an area of potential growth for pharmacy technicians in the US. An article from 2013 found that a pharmacy technician-centered medication reconciliation for ART therapy of patients in the hospital was successful in assisting with the prevention of drug-drug interactions, as well as other medication errors. The program showed that ART and OI prophylaxis in HIV/AIDS patients was improved by the utilization of pharmacy technicians [21]. Similar data were collected related to a pharmacy technician-centered medication reconciliation unit at a mental health location in 2014 [22]. These articles and pieces of literature show that there is potential for pharmacy technician-led integration of prevention of med errors even in disease-specific areas of healthcare.

In order to continue integrating pharmacy technicians into the practice of pharmacy, it is important for those pharmacy technicians to be highly skilled to enhance the clinical reach of the pharmacist. Projected pharmacy technician skills that could potentially be sought out by progressive employers include skills such as managing certain aspects of clinical tasks such as medication management and medication reconciliation, reiterating counseling points to reinforce statements made by the pharmacist, exceptional skills in communication and professionalism, as well as being able to quickly and accurately review information regarding patients’ prescriptions during data collection and order entry. One systematic review found that pharmacy technicians are often utilized to support MTM through medication reconciliation and that adherence and medication utility can be improved. However, standardization for administration utilization and educational training in this setting is necessary [23].

Another study found that the implementation of pharmacy technicians into a nursing team in an acute admissions unit in a hospital setting allowed for the prevention of omitted doses and helped all members of the team make better use of their time [24]. Another similar study showed that pharmacy technicians working in hospital wards in order to improve medication management and to prevent the utilization of expired or misplaced medications caused significant cost savings, as well as per-patient time savings for the nurses also working in the wards [25]. These and other studies like it allow us to see the benefit of utilizing pharmacy technicians in expanded definitions of the traditional pharmacy tech role. These articles and trials of expanding the role of pharmacy technicians have given insight into ways to continue utilizing pharmacy technicians well. Due to the vast amount of pharmacy technician positions in the US, and the variety in settings that pharmacy technicians can explore, it is expected that these potentially beneficial positions will continue to develop in the United States as pharmacy practice continues to move forward.

In addition to defining potential expansion of the role for technicians, the literature also emphasizes the importance of education and training in order to standardize patient care and to ensure best practice is being followed. Pharmacy technicians must be competent, able to communicate, and behave in a professional manner. Given their integral role in the profession of pharmacy, providing opportunities for pharmacy technicians to develop professionally is vital. Multiple professional organizations have provided outlines of competencies and training/education to assist pharmacy settings in providing these opportunities [1,13]. Opportunities also remain for technicians to become certified, and some jobs preferred or required this additional training [1,13]. However, the benefits of completing additional training for technicians may not yet be balanced with the costs of obtaining it [18].

### Limitations

This study, though novel, was limited in several ways. First, the available data were only taken from one source, Pharmacy Week. While Pharmacy Week does have a variety of job listings that encompass the entire United States, there are other sources of advertisements that are used to hire pharmacy technicians. The short data collection window only provides a brief snapshot of what jobs were being advertised during that time frame. While the sample size is robust for 14 days, it only reflects the needs at that specific time. Further, data collection was performed by four different researchers. While they all received training on the research protocol and data collection tools, no assessment for interrater reliability was performed.

## 5. Conclusions

This study represents a unique view of the state of pharmacy technician practice in the United States. Data showed demand for a broad variety of duties ranging from traditional to clinical to managerial. This represents an increased demand for skills and training requirements for pharmacy technicians. Further, due to the lack of standardization in certification and training for pharmacy technicians, it is important to research the future potential of policy, practice, and educational innovation in order to ensure safety and proper utilization of the pharmacy technician workforce.

## Figures and Tables

**Table 1:** Job Requirements Themes.

Job Requirements Themes	
Ability to perform tasks	Data input Intermediate calculations Technical skills Must be able to work in two specialty functions Clerical skills
Technology	Windows product proficiency Computer literacy Epic experience Use a 10-key pad
Communication	Language/communication (verbal, written, interpersonal) English proficiency Proficient in spoken Spanish Bilingual
Knowledge	Knowledge of metric system Special knowledge (drug names, Latin and chemical abbreviations, aseptic technique, storage of pharmaceuticals, determine drug’s formulary status) 340B experience Wholesale acquisition cost experience Medical terminology
Attributes	Reasoning/problem-solving skills Team player/leadership Customer service Detail oriented Time/work management (multitasking) Friendly/courtesy Flexibility Punctuality Professionalism Attendance Innovative Enthusiasm Salesman skills Ability to listen and learn Function in a normal work environment Function with minimal supervision Respect for confidentiality Strong ethical standards
Legal requirements	No previous drug use or conviction Subject to background and reference check Basic life support certification Have or get professional liability insurance U.S. citizen Driver’s license Selective service registration is required for males born after 12/31/1959 Up-to-date on vaccines
Physical	Physical demands (standing, lifting, bending) Visual acuity Auditory function
Age	18 years old or older 17 years old or older 16 years old or older
Previous experience	340B experience Wholesale acquisition cost (WAC) experience
Transportation	Reliable transportation Travel

**Table 2:** Job Duties by Theme.

Job Duties by Theme	
Do things under the direct supervision of the pharmacist	Do things under the direct supervision of the pharmacist Obtain a final check from the pharmacist before releasing any prepared parenteral compounds, before packaging any medication, or dispensing any medication
Communication	Triage requests and follow through with appropriate action(s) Notifies pharmacist of relevant clinical information gathered during calls to provider or patient that may affect the patient’s disease state or medication regimen Contacting insurance companies Provide information and assistance to pharmacies, members, and other callers regarding benefits, claims, and eligibility Read, interpret, and write documents (store and third-party clerical) Process faxes Ask if patient wants "pharmacist counseling" Refer any questions regarding prescriptions, drug information, or health matters to a pharmacist Notify pharmacist to transfer prescriptions that can no longer be filled to appropriate pharmacy along with notifying the provider and the patient Transcribe verbal prescriptions from doctors’ offices at the discretion of the pharmacist on duty Helping coordinate telehealth appointments
Perform pharmaceutical calculations	Calculate figures (mathematics) Calculate drug volumes to deliver correct dosages
Fill prescription orders	Receiving written prescription or refill requests and verify that information is complete and accurate Receives refill requests from patients and obtains authorization for refills from physicians’ offices Decipher and accurately enter orders for new prescriptions Billing/coding Identify and complete prior authorizations Prepare and distribute non-sterile medications Medication delivery (to home, pyxis...) Prescription counting, processing, filling, and labeling Maintain pharmacy records Pulling hard copy scripts to return to patient with an appropriate letter if pharmacy is unable to fill the order Ensure that patients receive the correct medication in a safe and timely manner
Fill IV medication orders	Prepare and distribute sterile medications Handles all home infusion functions as needed, including pump programming
Fill chemotherapy medication orders	Preparation of chemotherapy Demonstrates advanced knowledge of hazards of cytotoxic chemotherapeutic agents, including but not limited to the dangers posed to those who prepare, deliver, administer and/or receive treatment with these agents
Provider oversight of other employees	Oversight of other technicians Administrative task and staff support Coordinate technician activities (unit dose (UD) distribution, intravenous admixture, compounding, purchasing, controlled substances, OR drug preparation, pharmacy automation, investigational drug services, and inventory control) Assists in the supervision, scheduling, payroll maintenance, administration of disciplinary action, and evaluation of technical personnel Participates in recruitment activities and decisions to hire or terminate Provide and coordinate training
General office etiquette	Maintain clean work area Provide customer service Follow organization policies and procedures
Professionalism	Maintain personal appearance Work in a team (w/ other medical professionals) Participate in and successfully completes mandatory education Possess strong ethical standards Travel/attend meetings and conferences
Quality assurance/improvement	Develop and implement new systems and procedures Practice preventive maintenance by properly inspecting equipment and notify appropriate department or store manager of any items in need of repair Perform daily quality assurance monitoring/performance improvement activities Follow HIPPA standards for confidentiality Work with the pharmacist to ensure that the pharmacy functions and keeps within federal and state requirements Report medication diversions Report regulatory deficiencies (medication and billing errors) Notify the pharmacist when agents from any regulatory agency or law officers contact/visit the pharmacy. Assist with audits/work with auditing software Understand and adhere to guidelines on accepting and tendering vendor coupons, company limits on cash shortages and shrink guidelines. Participate in safety initiatives Follow United States Pharmacopeia (USP) standards (cleaning, PPE...) Inspect storage and maintain the safety of medications Assist in medication formulary management and compliance
Use of technology	Operate automated pharmacy technology systems Cash register operations Use computer system to credit unused doses back to patient accounts Use tools like a fork lift, hand tools, etc. Proficient in the use and application of new medications and technology Test client system
Inventory maintenance	Maintain/order inventory and supplies Manage the schedule for patient deliveries, manage inventory, create and maintain supply templates in the pharmacy computer database
Clinical tasks	Discusses with patients life issues affecting medication adherence and provide advice on improving drug regimen compliance Assist the pharmacist in medication reconciliation Reviews medication regimen for disease state and provide summaries and guidance on future medication plans. Advice may include alternate drug therapies, stopping a medication and/or lower cost alternative Help patients in over-the-counter (OTC) medication aisle Gather patient medication history Provide patient-oriented clinical pharmacy services to patients Provide care appropriate to the population served First-line screening for medication order errors, drug or allergy contraindications, and processing non-formulary drug requests Checking for possible interactions Assist patients in solving issues and problems related to acquired immunodeficiency syndrome (AIDS)
Maintain workflow in a high-volume pharmacy	
HIV knowledgeable	
Maintain narcotic coordination and investigational drug therapy	
Perform duties of a technician	
Business configuration duties	
Provides PAP assistance	
Promotion of services	Set up and maintain pharmacy display cases Be aware of competitor services and effectiveness Promote company services to obtain new customers
Prepare, distribute, and maintain records for investigational drug products ensuring that you understand study protocols needed to accurately fulfill orders	
Research how the pharmacy can acquire contracts for certain state Medicaid’s/Adaps/Networks, depending on the needs at the moment	
Pick-up orders, requisitions, and medications when on delivery rounds	
Subject matter expert of delivery services and leader of delivery initiatives	
Opening, counting, barcoding, and profiling incoming mail	
Manages difficult or emotional patient situations	
Other duties as assigned to include	This job description is not intended, nor should it be construed to be an exhaustive list of all responsibilities, skills, efforts or working conditions associated with the job. It is intended to indicate the general nature and level of work performed by employees within this classification. Other duties as assigned

**Table 3:** Pharmacy Technician Job Requirements.

Requirement	Required Number (%)	Preferred Number (%)	Not Required Number (%)	Not Specified Number (%)
Registration with State Board	18,261 (86.9%)	16 (0.1%)	102 (0.5%)	2628 (12.5%)
High school diploma	17,325 (82.5%)	69 (0.3%)	116 (0.6%)	3497 (16.6%)
Ability to perform tasks	16,861 (80.3%)	-	-	4146 (19.7%)
Communication	16,436 (78.2%)	-	-	4571 (21.8%)
Physical	15,908 (75.7%)	-	-	5099 (24.3%)
Technology	10,132 (48.2%)	-	-	10,875 (51.8%)
Attributes	6478 (30.8%)	-	-	14,529 (69.2%)
PTCB or exCPT certification	2583 (12.3%)	17,114 (81.5%)	4 (0%)	1306 (6.2%)

PTCP = Pharmacy Technician Certification Board; exCPT = Exam for the Certification of Pharmacy Technicians.

**Table 4:** Pharmacy Technician Job Duties.

Duty	Listed Number (%)	Not Listed Number (%)
General office etiquette	19,961 (95%)	1046 (5%)
Do things under the direct supervision of the pharmacist	18,043 (85.9%)	2964 (14.1%)
Professionalism	10,560 (50.3%)	10,447 (49.7%)
Fill prescription orders	10,079 (48%)	10,928 (52%)
Quality assurance and improvement	8870 (42.2%)	12,137 (57.8%)
